# Mycoplasma pneumoniae‐associated Fuchs syndrome

**DOI:** 10.1002/ccr3.1350

**Published:** 2017-12-22

**Authors:** Babikir Kheiri, Nour Aljariri Alhesan, Seetharamprasad Madala, Omar Assasa, Meng Shen, Thair Dawood

**Affiliations:** ^1^ Internal Medicine Department Hurley Medical Center/Michigan State University Two Hurley Plaza, Ste 212 Flint 48503 Michigan

**Keywords:** Atypical Stevens–Johnson syndrome, Fuchs syndrome, Mycoplasma pneumonia and mucositis, Mycoplasma pneumonia‐induced rash and mucositis

## Abstract

Fuchs syndrome is a milder form of the Stevens–Johnson syndrome (SJS) spectrum with only mucosal involvement which can be triggered by Mycoplasma pneumonia (MP) infection. Treatment should be directed toward supportive care including ocular and mucous membrane care, fluids and nutritional support, and pain control. In addition, antibiotic and immunomodulatory treatments are discussed for this entity.

## Quiz Question

What is the diagnosis and what is the treatment?

**Figure 1:**
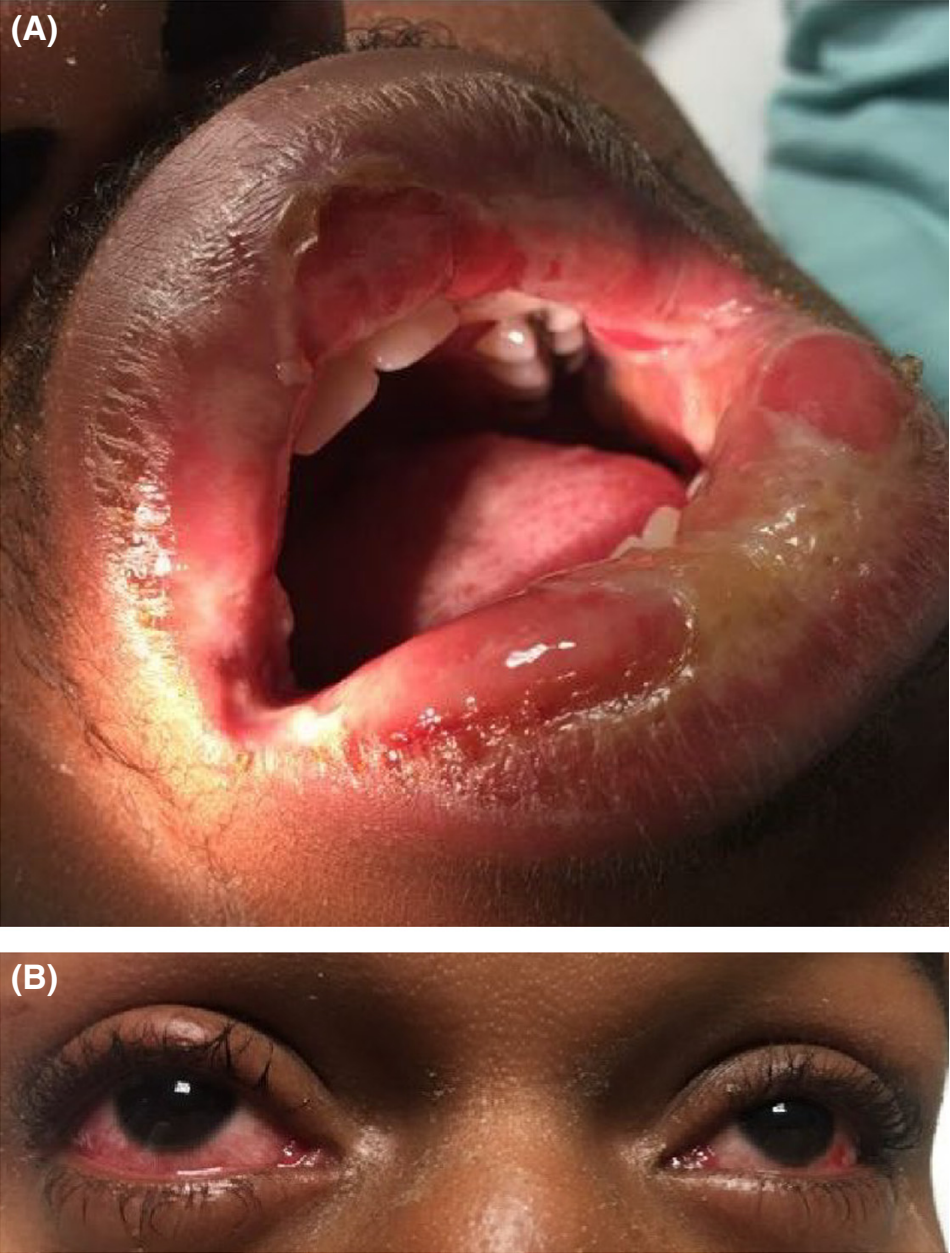
(A) Mucositis and erythematous lips erosions. (B) Bilateral conjunctival injection.

A 20‐year‐old man was admitted with 2‐week history of bilateral eye redness and painful oral mucosal ulcerations. He denied any respiratory symptoms. Physical examination was notable for fever (39.5), bilateral conjunctival injection with mucositis and erythematous lips erosions (Fig. 1A and B). Chest examination was unremarkable, and there were no genital lesions. Laboratory investigations showed white cell count 17.5 (Ref: 4.5–13.5 K/UL) predominately neutrophils 82% (Ref: 36–75%), high‐sensitive C‐reactive protein 17.021 (Ref: <0.3 MG/DL), and positive MP IgG 1.16 and MP IgM 2.47 (Ref: ≤0.90 INDEX). Chest X ray (CXR) was unremarkable.

Supportive treatment was initiated with intravenous (IV) fluids to maintain hydration. In addition, he received oral azithromycin 500 mg for 5 days and 3 days of 1 g intravenous (IV)‐pulse‐methylprednisolone followed by 2 days of 80 mg IV‐methylprednisolone and then oral prednisone 60 mg. Total parenteral nutrition (TPN) was given initially to support nutrition, which was subsequently stopped as he tolerated oral feeds. He made full recovery within 10 days and was discharged home in stable condition on tapering doses of prednisone.

Mycoplasma pneumonia (MP) can be associated with isolated mucous membrane disease with or without minimal skin lesions and referred to “atypical Stevens–Johnson syndrome (SJS), Fuchs syndrome, M. pneumonia‐associated mucositis (MPAM), or MP‐induced rash and mucositis (MIRM)” 1, 2. This condition falls into the epidermolysis spectrum of erythema multiform/SJS 2 and may have a milder disease course with rare long‐term sequelae and mortality. Supportive care is the main stay of treatment, and the role of antibiotics, steroids, and/or immunoglobulins is limited with no agreed evidence‐based guidelines 1.

## Authorship

BK: designed, planned, wrote the manuscript and did the literature review. NA: designed, planned, and revised the manuscript. SM: designed, planned, revised the manuscript and prepared the photographs. OA: designed, planned, revised the manuscript and edited the photographs. MS: designed, planned, revised the manuscript. TD: designed, planned, revised the manuscript and selected the photographs.

## Conflict of Interest

None declared.
